# Branching patterns of the afferent branchial arteries and their phylogenetic significance in rays (Batoidea)

**DOI:** 10.1038/s41598-021-02145-x

**Published:** 2021-12-01

**Authors:** Karla D. A. Soares, Mônica Toledo-Piza

**Affiliations:** Departamento de Zoologia, Instituto de Biociências, Universidade de São Paulo, Rua do Matão, Trav. 14, no. 101, São Paulo, SP CEP 05508-090 Brazil

**Keywords:** Phylogenetics, Ichthyology

## Abstract

Rays of the superorder Batoidea comprise the most diverse group of chondrichthyans in terms of valid species and morphological disparity. Up to the present little agreement is observed in studies based on morphological and molecular data focused on uncovering the interrelationships within Batoidea. Morphology-based phylogenies of batoids have not included characters related to the afferent branchial arteries, and little is known about the variation in this anatomical complex in rays. Herein, representatives of 32 genera from 19 families currently recognized of rays were examined as well as some shark taxa. Seven new characters are proposed and tested in two different analyses, one on their own and in the other they were added to the morphological data matrix of the most recent analysis of interrelationships within Batoidea. The arrangement of afferent branchial arteries differs mainly among orders and families of batoids. The absence of a common trunk from which the three posteriormost afferent arteries branch is interpreted as a synapomorphy for Myliobatiformes and the presence of a coronary cranial artery as an autapomorphy for *Mobula hypostoma*. A close spatial relationship between the second and third afferent arteries within the common branch from the ventral aorta is proposed as a synapomorphy for Rajiformes with a secondary modification in *Sympterygia*. Data about patterns in afferent branchial arteries in additional taxa such as Squaliformes and Chimaeriformes are needed to better understand the evolution of this character complex among chondrichthyans.

## Introduction

The superorder Batoidea comprises 640 valid species of skates, stingrays and close allies (sawfishes, guitarfishes, electric rays) and the monophyly of this taxon has been corroborated by previous studies using morphological and molecular data^1–13^. On the other hand, the phylogenetic position of rays among chondrichthyans and consequently elasmobranch inter-relationships are still the subject of intense debate. Analyses based on morphological characters have reunited rays, sawsharks and angel sharks into the putatively monophyletic taxon Hypnosqualea, a highly derived clade within squalomorph sharks^2,7,8^ whereas recent phylogenetic hypotheses using molecular data place rays as the sister-group of all remaining elasmobranchs^4–6,9–15^.

Hypotheses regarding the phylogenetic relationships within Batoidea are also controversial. Differences regarding the number of recognized supraspecific taxa as well as the definition of morphological characters and their states have led to divergent hypotheses among morphology-based phylogenetic studies^1–3,7,8,16–22^. Compagno^1^ hypothesized the Torpediniformes (electric rays) as the sister group of all other extant batoids based on the “generalized or typical chondrichthyan ventral gill arch structure” a hypothesis supported by the studies of McEachran et al.^22^, McEachran & Aschliman^20^ and Aschliman et al.^3^. However, Shirai^2,7^ placed Pristiformes and not the Torpediniformes as the most basal group within batoids. Alternatively, analyses including molecular^4–6,11,12,23^ and karyological^13^ data hypothesized either the Rajiformes, or Torpediniformes, or Rhinobatiformes as the sister group of all remaining batoids. In addition, conflicting hypotheses persist regarding the placement of the guitarfishes belonging to the families Plathyrhinidae and Zanobatidae^1,3–6^. Different classifications have already been proposed for batoid taxa and more recent molecular studies divided batoids into four orders (Rajiformes, Torpediniformes, Rhinopristiformes and Myliobatiformes)^5^.

The derived condition of batoid skeletal features such as the amphistylic jaw articulation and the presence of a synarcual is widely documented in the literature^1–3,17,24^ while systematic studies based on characters of soft tissues are less numerous^25,26^. There are detailed descriptions in the classical literature of the heart morphology and its related blood vessels in various elasmobranchs (e.g.^27–38^), however they are usually focused on a single species. More recent studies included more representatives (e.g.^39–43^), and some of them reported significant variation. For instance, Muñoz-Chápuli et al.^25^ examined the arrangement of coronary arteries and proposed a derived condition shared between rajoids and myliobatoids (= Rajiformes and Myliobatiformes, respectively). Nevertheless, phylogenetic analyses of batoids based on morphology have not included characters related to the branching patterns of afferent branchial arteries^3,17–22^.

Herein, morphological variation in afferent branchial arteries across batoids and some shark representatives are described in detail, characters are proposed and tested within a phylogenetic framework. The present study aims to improve our understanding of the branching patterns of the branchial afferent arteries and their phylogenetic significance within Batoidea.

## Results

### Character description

The heart is situated in the pericardial cavity, dorsal and anterior to the ventrally located symphysis of the right and left shoulder girdle and ventral to the pharynx. From the *conus arteriosus* of the heart, the ventral aorta extends anteriorly and from it the afferent branchial arteries arise either separately or through common trunks. These vessels are covered ventrally by the hypobranchial musculature and are situated ventral to the hyoid and gill arches.

**Figure 1:**
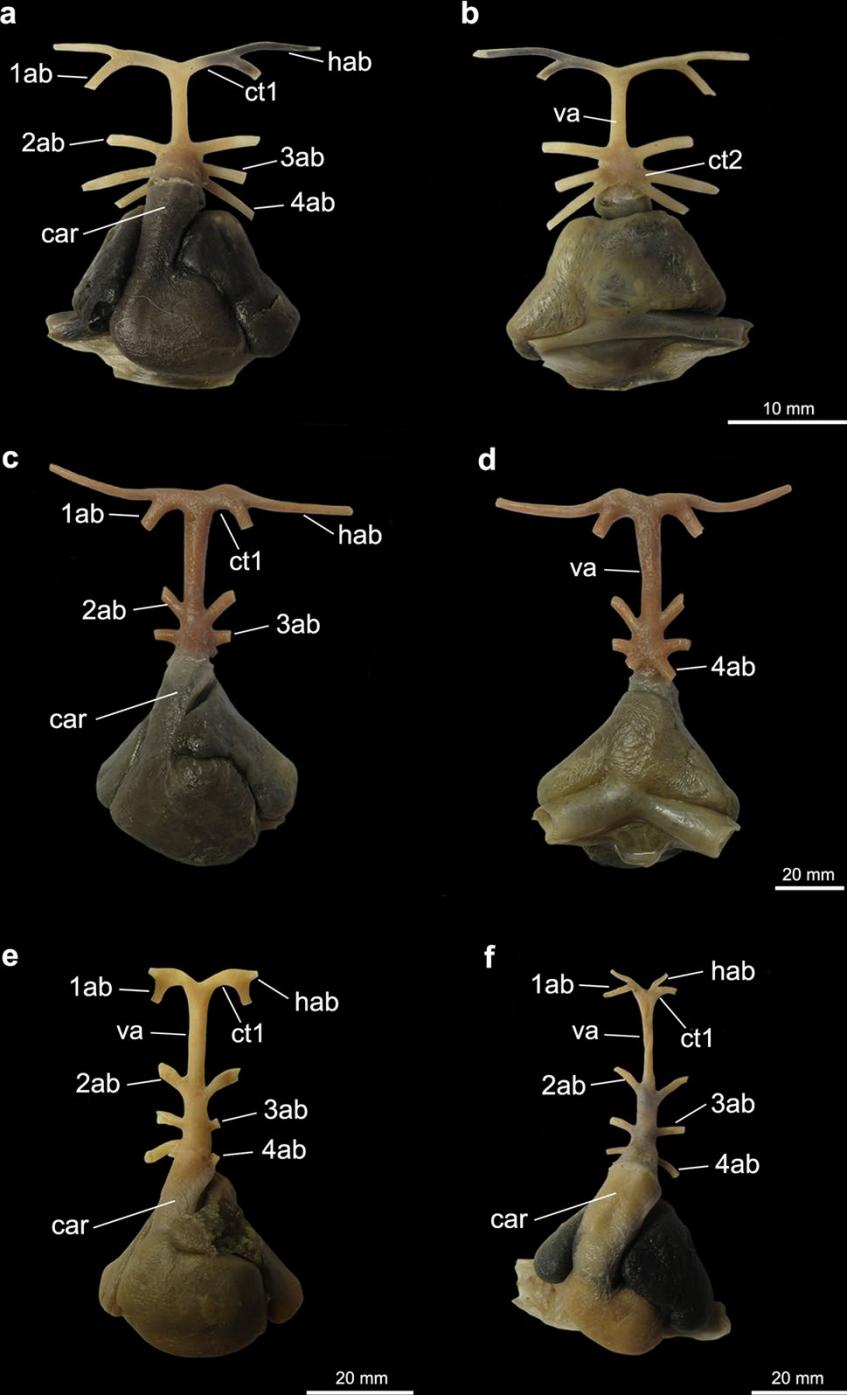
Heart and afferent branchial arteries in Myliobatiformes. (**a**) ventral and (**b**) dorsal views of *Gymnura micrura*; (**c**) ventral and (**d**) dorsal views of *Myliobatis freminvillei*; (**e**) ventral view of *Urobatis halleri*; (**f**) ventral view of *Potamotrygon motoro*. *1ab-4ab* 1st-4th afferent branchial arteries; *ct1* common trunk from the ventral aorta to the hyoidean and first afferent branchial arteries; *car* *conus arteriosus*; *hab* hyoidean afferent branchial artery; *ct2* common trunk from the ventral aorta to the two posteriormost afferent branchial arteries; *va* ventral aorta.

**Figure 2:**
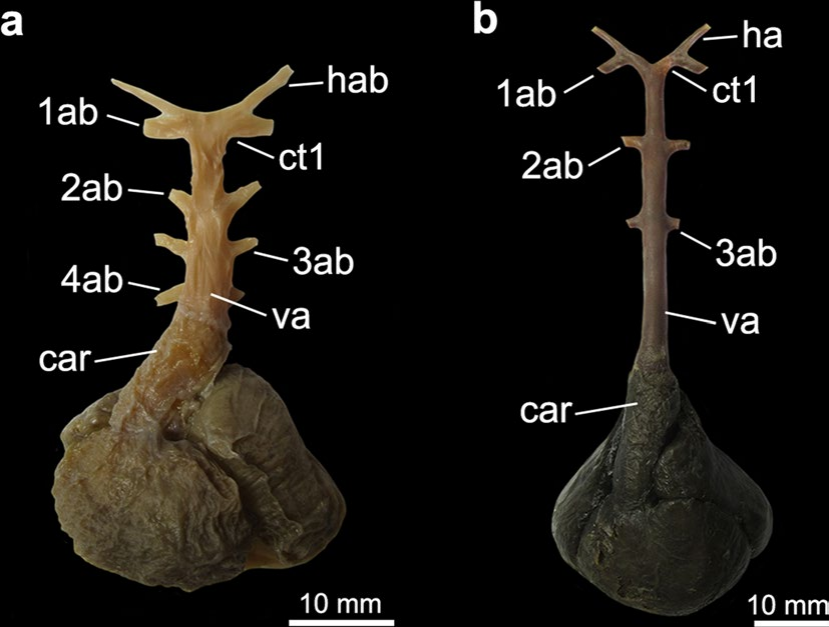
Heart and afferent branchial arteries in Myliobatiformes. (**a**) Ventral view of *Rhinoptera bonasus*; (**b**) ventral view of *Mobula hypostoma*, fourth branchial afferent artery not shown. *1ab-4ab* 1st-4th afferent branchial arteries; *car conus arteriosus*; *ct1* common trunk from the ventral aorta to the hyoidean and first afferent branchial arteries; *hab* hyoidean afferent branchial artery; *va* ventral aorta.

**Figure 3:**
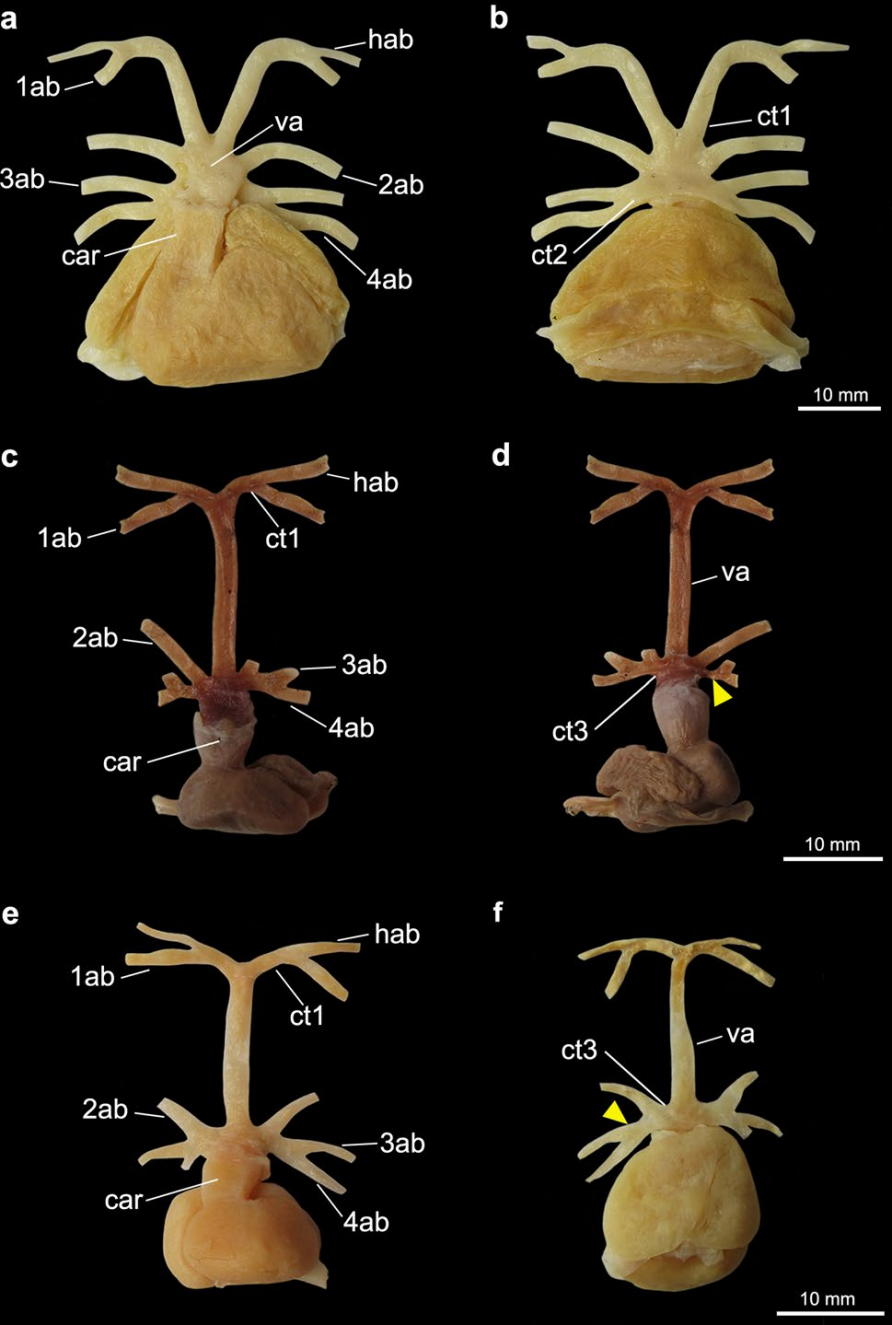
Heart and afferent branchial arteries in Torpediniformes. (**a**) ventral and (**b**) dorsal views of *Tetronarce puelcha*; (**c**) ventral and (**d**) dorsal views of *Narcine brasiliensis*; (**e**) ventral and (**f**) dorsal views of *Heteronarce* sp. *1ab-4ab* 1st-4th afferent branchial arteries; *car* *conus arteriosus*; *ct1* common trunk from the ventral aorta to the hyoidean and first afferent branchial arteries; *ct2* common trunk from the ventral aorta to the two posteriormost afferent branchial arteries; *ct3* common trunk from the ventral aorta to the three posteriormost afferent branchial arteries; *hab* hyoidean afferent branchial artery; *va* ventral aorta. Yellow triangles point to the common vessel that originates the third and fourth afferent branchial arteries.

**Figure 4:**
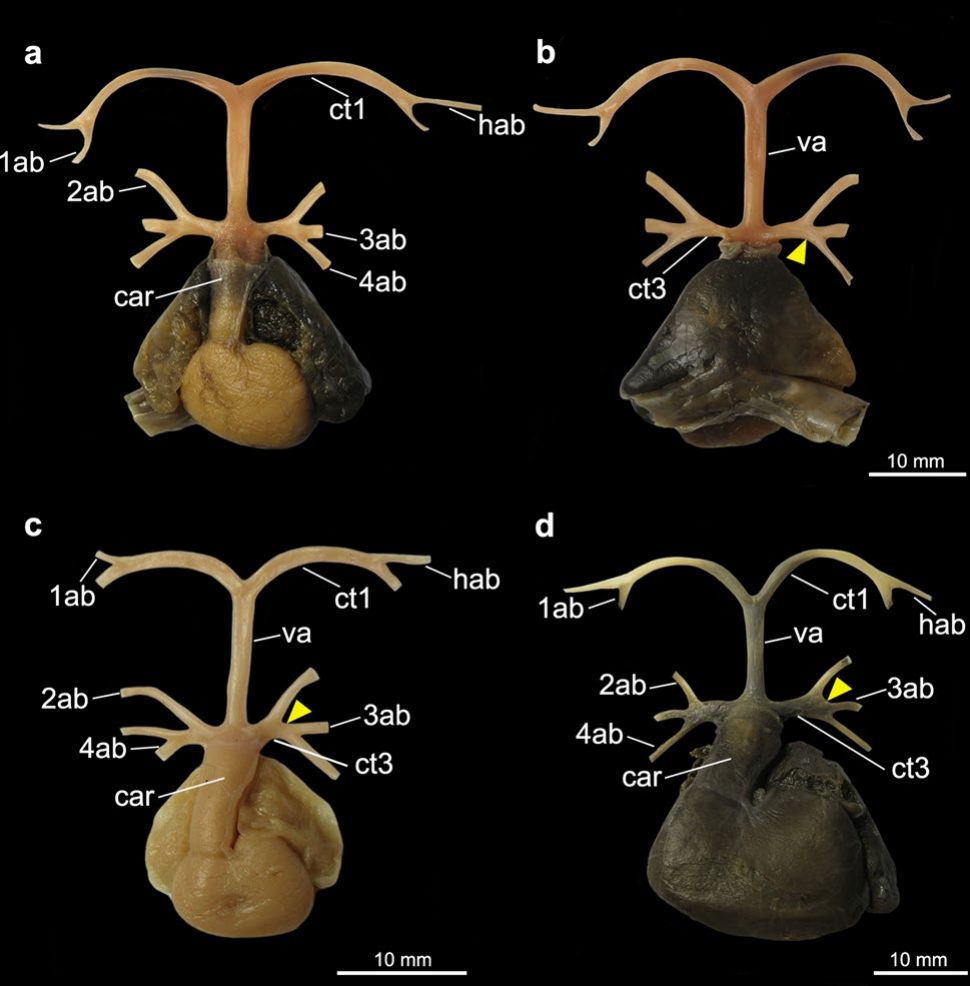
Heart and afferent branchial arteries in Rhinobatiformes and Rhiniformes. (**a**) ventral and (**b**) dorsal views of *Pseudobatos horkelii;* (**c**) ventral view of *Glaucostegus granulatus*; (**d**) ventral view of *Rhynchobatus palpebratus*, *1ab-4ab* 1st-4th afferent branchial arteries; *car conus arteriosus*; *ct1* common trunk from the ventral aorta to the hyoidean and first afferent branchial arteries; *ct3* common trunk from the ventral aorta to the three posteriormost afferent branchial arteries; *hab* hyoidean afferent branchial artery; *va* ventral aorta. Yellow triangles point to the common vessel that originates the third and fourth afferent branchial arteries.

**Figure 5:**
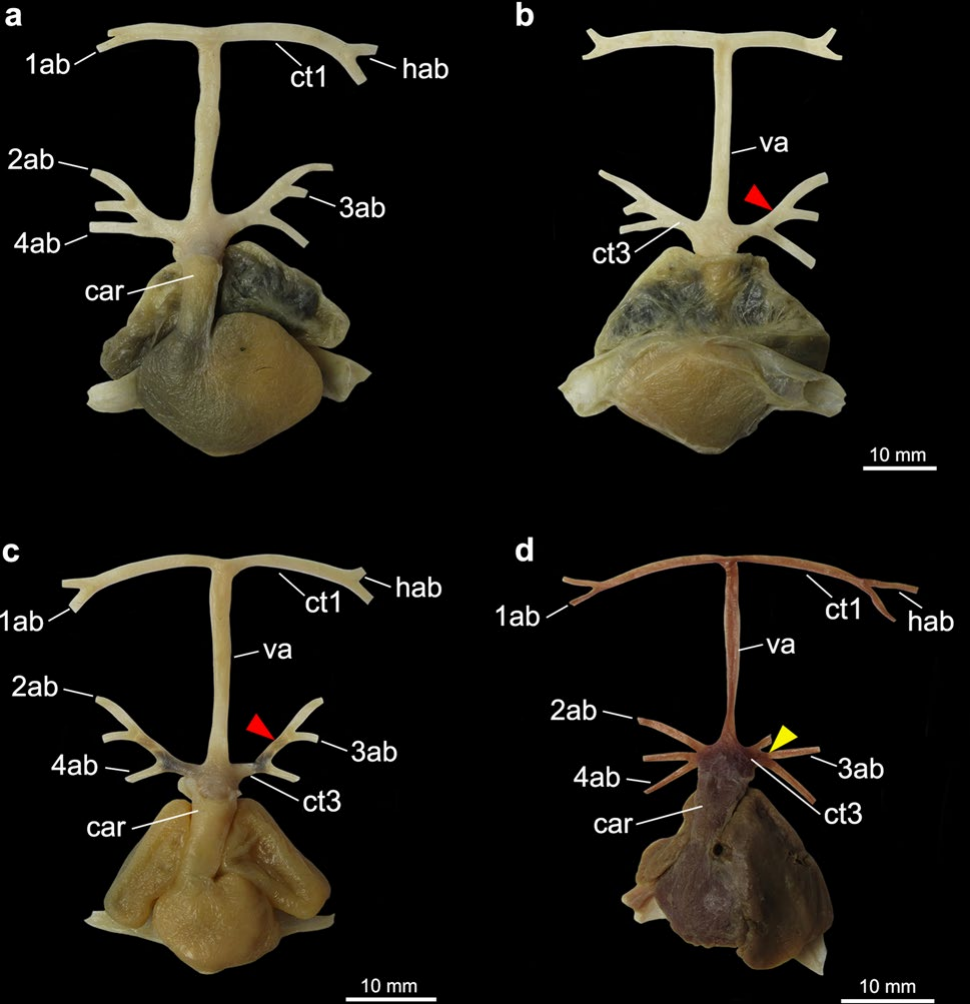
Heart and afferent branchial arteries in Rajiformes. (**a**) ventral and (**b**) dorsal views of *Atlantoraja cyclophora*; (**c**) ventral view of *Rioraja agassizii*; (**d**) ventral view of *Sympterygia acuta*. *1ab-4ab* 1st-4th afferent branchial arteries; *car* *conus arteriosus*; *ct1* common trunk from the ventral aorta to the hyoidean and first afferent branchial arteries; *ct3* common trunk from the ventral aorta to the three posteriormost afferent branchial arteries; *hab* hyoidean afferent branchial artery; *va* ventral aorta. Red triangles point to the common vessel that originates the second and third arteries and yellow one to the common vessel to third and fourth afferent branchial arteries.

**Figure 6:**
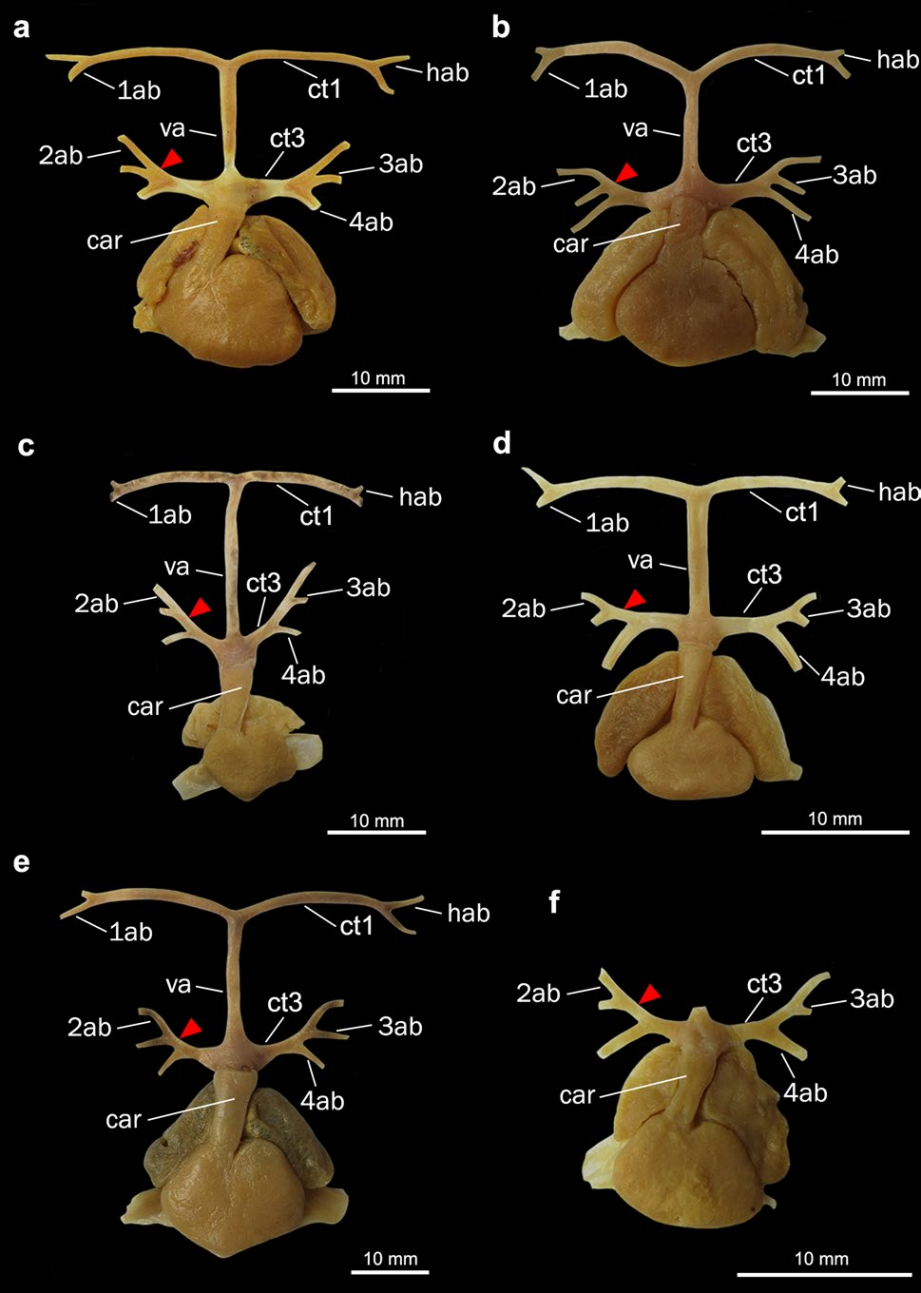
Heart and afferent branchial arteries in Rajiformes. (**a**) ventral view of *Rajella purpuriventralis*; (**b**) ventral view of *Zearaja chilensis*; (**c**) ventral view of *Raja miraletus*; (**d**) ventral view of *Malacoraja senta*; (**e**) ventral view of *Dipturus* sp.; (**f**) ventral view of *Cruriraja rugosa*, ventral aorta and two anteriormost afferent arteries not shown. *1ab-4ab* 1st-4th afferent branchial arteries; *car* *conus arteriosus*; *ct1* common trunk from the ventral aorta to the hyoidean and first afferent branchial arteries; *ct3* common trunk from the ventral aorta to the three posteriormost afferent branchial arteries; *hab* hyoidean afferent branchial artery; *va* ventral aorta. Red triangles point to the common vessel that originates the second and third afferent branchial arteries.

**Figure 7:**
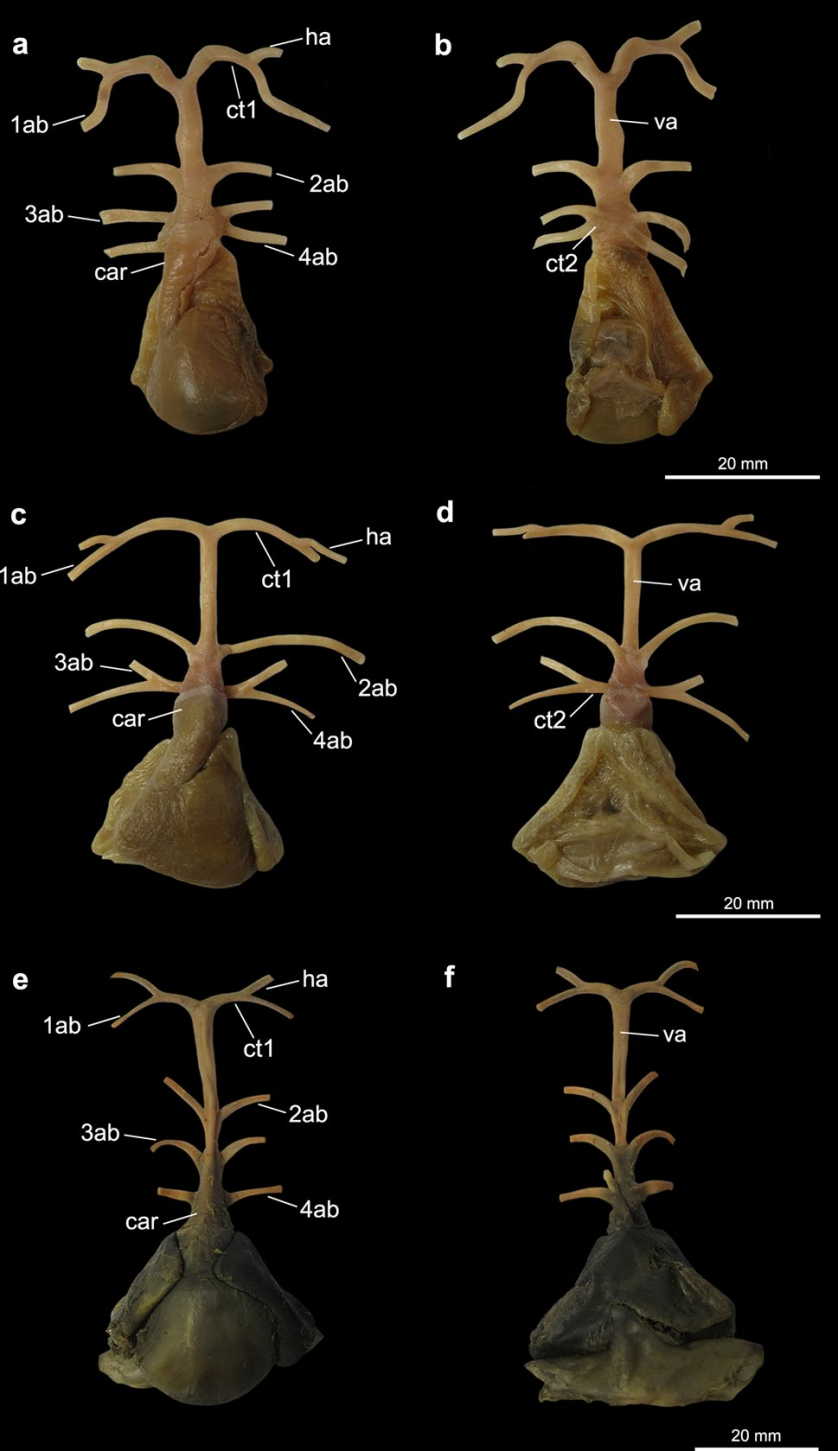
Heart and afferent branchial arteries in shark specimens. (**a**) Ventral and (**b**) dorsal views of *Squalus* sp.; (**c**) ventral and (**d**) dorsal views of *Squatina occulta*; (**e**) ventral and (**f**) dorsal views of *Negaprion brevirostris*. *1ab-4ab* 1st-4th afferent branchial arteries; *car* *conus arteriosus*; *ct1* common trunk from the ventral aorta to the hyoidean and first afferent branchial arteries; *ct2* common trunk from the ventral aorta to the two posteriormost afferent branchial arteries; *hab* hyoidean afferent branchial artery; *va* ventral aorta.

**Figure 8:**
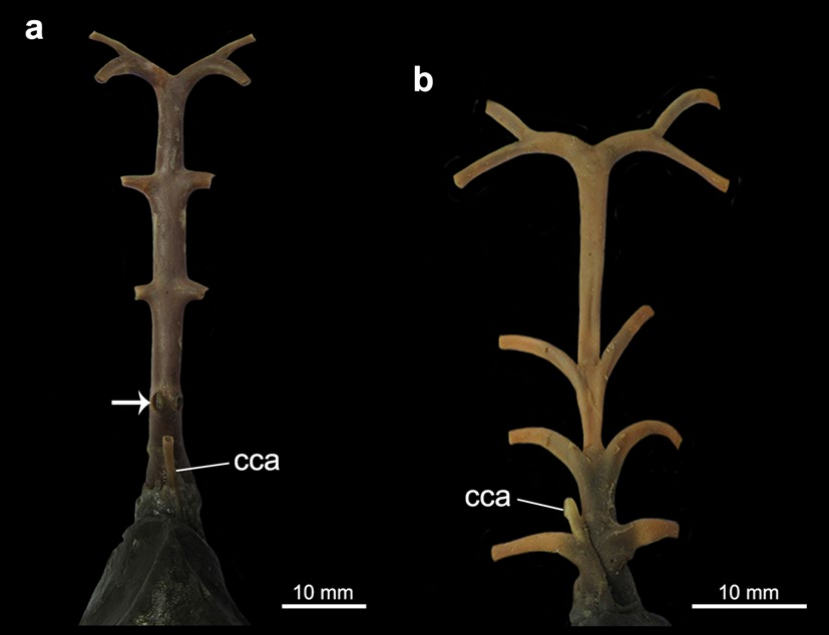
Dorsal view of the region of ventral aorta and afferent branchial arteries of (**a**) *Mobula hypostoma* and (**b**) *Negaprion brevirostris*. Arrow points to the opening of the fourth afferent branchial artery. *cca* cranial coronary artery.

All batoids and sharks examined present a similar branching pattern for the hyoidean and the first afferent, which split from a common trunk that originates at the anterior end of the ventral aorta (Figs. 1, 2, 3, 4, 5, 6, 7 and 8). Variation in the vessels of this region among taxa was observed in the relative length of the common trunk of the hyoidean and first afferent branchial arteries; in the relative length of the ventral aorta; and in the branching pattern of the three posteriormost afferent branchial arteries, those associated with branchial arches 2, 3 and 4. Considering the variation observed, seven characters and their respective states are distinguished and discussed below (Table 1).

**Table 1:** List of batoid and shark species examined in the present study with respective character states from afferent branchial arteries.

Order	Family	Species examined	Characters						
			1	2	3	4	5	6	7
Carcharhiniformes	Carcharhinidae	*Negaprion brevirostris*	0	0	0	0	–	–	1
	Scyliorhinidae	*Scyliorhinus haeckelii*	0	0	1	0	0	–	0
Squaliformes	Squalidae	*Squalus* sp.	1	0	1	0	0	–	0
Squatiniformes	Squatinidae	*Squatina occulta*	1	0	1	0	1	–	0
Myliobatiformes	Dasyatidae	*Hypanus marianae*	0	0	0	0	–	–	0
	Gymnuridae	*Gymnura micrura*	0	0	1	0	0	–	0
	Mobulidae	*Mobula hypostoma*	0	0	0	0	–	–	1
	Myliobatidae	*Myliobatis freminvillei*	0	0	0	0	–	–	0
	Potamotrygonidae	*Potamotrygon motoro*	0	0	0	0	–	–	0
	Rhinopteridae	*Rhinoptera bonasus*	0	0	0	0	–	–	0
	Urotrygonidae	*Urobatis halleri*	0	0	0	0	–	–	0
Rajiformes	Anacanthobatidae	*Schroederobatis americana*	1	0	0	1	–	1	0
	Arhynchobatidae	*Atlantoraja cyclophora*	1	0	0	1	–	1	0
		*Psammobatis extenta*	1	0	0	1	–	1	0
		*Rioraja agassizii*	1	0	0	1	–	1	0
		*Sympterygia acuta*	1	0	0	1	–	1	0
		*S. bonapartii*	1	0	0	1	–	1	0
	Gurgesiellidae	*Cruriraja rugosa*	1	0	0	1	–	1	0
		*Gurgesiella atlantica*	1	0	0	1	–	1	0
	Rajidae	*Dactylobatus clarkii*	1	0	0	1	–	1	0
		*Dipturus* sp.	1	0	0	1	–	1	0
		*Leucoraja garmani*	1	0	0	1	–	1	0
		*Malacoraja senta*	1	0	0	1	–	1	0
		*Raja miraletus*	1	0	0	1	–	1	0
		*Rajella purpuriventralis*	1	0	0	1	–	1	0
		*Zearaja chilensis*	1	0	0	1	–	1	0
Rhiniformes	Rhinidae	*Rhynchobatus palpebratus*	1	0	0	1	–	0	0
Rhinobatiformes	Glaucostegidae	*Glaucostegus granulatus*	1	0	0	1	–	0	0
	Rhinobatidae	*Pseudobatos horkelii*	1	0	0	1	–	0	0
	Trygonorhinidae	*Zapteryx brevirostris*	1	0	0	1	–	0	0
Torpediniformes	Narcinidae	*Benthobatis kreffti*	0	0	0	1	–	0	0
		*Discopyge tschudii*	0	0	0	1	–	0	0
		*Narcine brasiliensis*	0	0	0	1	–	0	0
	Narkidae	*Heteronarce* sp.	0	0	0	1	–	0	0
		*Temera hardwickii*	0	0	0	1	–	0	0
	Torpedinidae	*Tetronarce puelcha*	1	1	1	0	0	–	0
*Incertae sedis*	Platyrhinidae	*Platyrhinoidis triseriata*	1	0	0	1	–	0	0

#### Character 1

Relative length of the common trunk that then branches into hyoidean and first afferent branchial arteries: (0) short, with distal end near proximal end, (1) long, with distal end widely separated from proximal end.

In the Myliobatiformes and the Torpediniformes (except *Tetronarce puelcha*), the branching point of the hyoidean and first afferent branchial arteries is very close to the anterior tip of the ventral aorta (state 0; Figs. 1c–f, 2, 3c–f), so that the relative length of the common vessel from the ventral aorta to those afferent arteries is considerably shorter when compared to the condition present in Rajiformes, guitarfishes, *Platyrhinoidis triseriata* and *Tetronarce puelcha* (state 1; Figs. 4, 5, 6). In *Gymnura* (Fig. 1a,b), the common vessel that then branches into the hyoidean and first afferent branchial arteries is longer than that of other Myliobatiformes examined. However, it is still shorter than the same common vessel in Rajiformes, *Platyrhinoidis triseriata* and *Tetronarce puelcha*. It was not possible to unequivocally assign a character state to express this condition in *Gymnura*, so we tentatively coded it as state ‘0’. Although the trunk from the ventral aorta to the two anteriormost afferent arteries has a different orientation in *Tetronarce puelcha* when compared to the other taxa in which this trunk is also long, it is clearly longer than the condition of the other Torpediniformes and Myliobatiformes. Among the sharks examined, in *Negaprion* (Fig. 7e,f) and *Scyliorhinus*, this trunk is short (state 0), and in *Squatina* and *Squalus* it is clearly longer (state 1; Fig. 7a–d).

#### Character 2

Relative distance between the anterior tip of the ventral aorta and the anterior end of the *conus arteriosus*: (0) long, extending anteriorly to some extent as a single vessel, (1) short, with the base and anterior tip of ventral aorta next to each other.

In all examined batoids (except *Tetronarce puelcha*), the ventral aorta runs anteriorly to some extent as a single vessel, from the base of the *conus arteriosus* to the branching of the common trunk of the hyoidean and first afferent branchial arteries, so that the base and the anterior tip of the ventral aorta are relatively well separated. In contrast, in *Tetronarce puelcha* the relative distance between the anterior tip of the ventral aorta and the anterior end of the *conus arteriosus* is very short, so that the branching points of all vessels from the ventral aorta are next to each other (state 1; Fig. 3a,b). In the illustration of *Torpedo californica* (= *Tetronarce californica*) provided by Miyake et al.^26^ the arrangement of those vessels is similar to that in *Tetronarce puelcha*. Satchell^42^ (p. 220, Fig. 9.1B) also illustrated a condition in *Torpedo fairchildi* (= *Tetronarce nobiliana*) that is somewhat similar to that of *T. puelcha*; however, he did not describe the condition any further. In all examined sharks the ventral aorta is relatively long, a condition similar to that examined in most batoids (state 0; Fig. 7).

#### Character 3

Common trunk off the ventral aorta for the third and fourth afferent branchial arteries: (0) absent, (1) present.

Among all batoids examined, only in *Gymnura* and *Tetronarce* the third and fourth afferent branchial arteries arise from a common trunk that originates from the ventral aorta, (state 1). Despite being relatively short, this common stem is present and more evident in a dorsal view (Figs. 1b, 3b). Although the origins of the third and fourth afferent branchial arteries in *Myliobatis* are very close to each other, they still branch independently from the ventral aorta, a condition also observed by Kobelkowsky^43^. In all remaining batoids, a common trunk off the ventral aorta for third and fourth afferent branchial arteries is absent (state 0).

Among the sharks examined, *Scyliorhinus, Squalus* and *Squatina* have the third and fourth afferent branchial arteries branching from a common vessel that originates from the ventral aorta while in *Negaprion* those arteries originate independently from the ventral aorta (Fig. 7). O’Donogue and Abbot^37^ reported that there is variation of this condition in *Squalus acanthias*, with the presence of a common trunk to the third and fourth arteries originating from the ventral aorta in this species being relatively rare, and the most common condition is that in which the two posteriormost arteries are very close together at their bases, but still originate independently from the ventral aorta. All four specimens of *Squalus* examined herein have the third and fourth afferent arteries originating from a common vessel that originate from the ventral aorta (Fig. 7a) and a similar condition was also illustrated in previous works^42,46^.

#### Character 4

Common trunk off the ventral aorta branching into the second, third and fourth afferent branchial arteries: (0) absent, (1) present.

In all examined Rajiformes, guitarfishes, *Platyrhinoidis triseriata* and the Torpediniformes (except *Tetronarce*), the second, third and fourth afferent branchial arteries branch from a common trunk off the ventral aorta (state 1; Figs. 3c–f, 4, 5, 6). In all examined Myliobatiformes and in *Tetronarce puelcha*, the second afferent branchial artery branches directly from the ventral aorta, independently from the last two afferent arteries (state 0; Figs. 1, 2, 3a,b). The same condition occurs in the examined shark specimens (Fig. 7).

#### Character 5

Relative length of the trunk off the ventral aorta for the third and fourth afferent branchial arteries: (0) short, with branching point into the third and fourth afferent arteries close the origin on ventral aorta, (1) long, with branching point into the third and fourth afferent arteries distinctly separated from origin on ventral aorta.

The trunk from the ventral aorta for the third and fourth afferent branchial arteries in the myliobatiform *Gymnura* and the torpediniform *Tetronarce puelcha* is relatively short so that the branching point of the two last afferent arteries is close to the ventral aorta (state 0; Figs. 1a,b, 3, 5d). Among the shark species examined, *Scyliorhinus* and *Squalus* have a similar condition, whereas in *Squatina* (Fig. 7) the common trunk for the third and fourth arteries is longer, with the branching point of the afferent arteries distinctly farther from the ventral aorta. All remaining taxa, that do not possess a trunk off the ventral aorta that branches into the third and fourth afferent arteries were coded as ‘–’.

#### Character 6

Branching pattern of second, third and fourth afferent branchial arteries from the common trunk of the ventral aorta: (0) third and fourth arteries splitting from a common branch, (1) second and third arteries splitting from a common branch.

Among taxa in which the second, third and fourth afferent arteries branch from the common trunk of the ventral aorta, there is difference in the branching pattern of these vessels relative to one another. In Rajiformes (except *Sympterygia*) the second and third afferent arteries split from a common branch (state 1; Figs. 5a–c, 6), while in *Sympterygia, Platyrhinoidis triseriata*, guitarfishes, the torpediniforms Narcinidae and Narkidae, the third and fourth arteries split from a common branch (state 0; Figs. 3c–f, 4, 5d).

Among species in which the second and third afferent arteries split from a common branch (i*.e*., all Rajiformes except *Sympterygia*), there is variation in the relative length of this common branch: in *Gurgersiella atlantica*, *Rajella purpuriventralis*, *Schroederobatis americana* and *Zearaja chilensis*, it is relatively shorter than of other skates (compare Figs. 6a,b,e,f). Nevertheless, we did not propose a distinct character for this feature because it was not possible to unambiguously define discrete states for the variation observed.

#### Character 7

Cranial coronary artery: (0) absent, (1) present.

Coronary arteries are part of the coronary circulation and most of them are derived cranially from the hypobranchial artery that approaches the heart along the dorsal surface of the *conus arteriosus*^49,50^. They can be divided in cranial and caudal units, providing blood with higher oxygen tensions directly to the heart^51^. A well-developed cranial coronary artery is found in *Mobula hypostoma* and *Negaprion brevirostris* (state 1; Fig. 8). A cranial coronary artery is absent in the other taxa examined. Caudal coronary supplies were not examined in this study.

### Phylogenetic analyses

**Figure 9:**
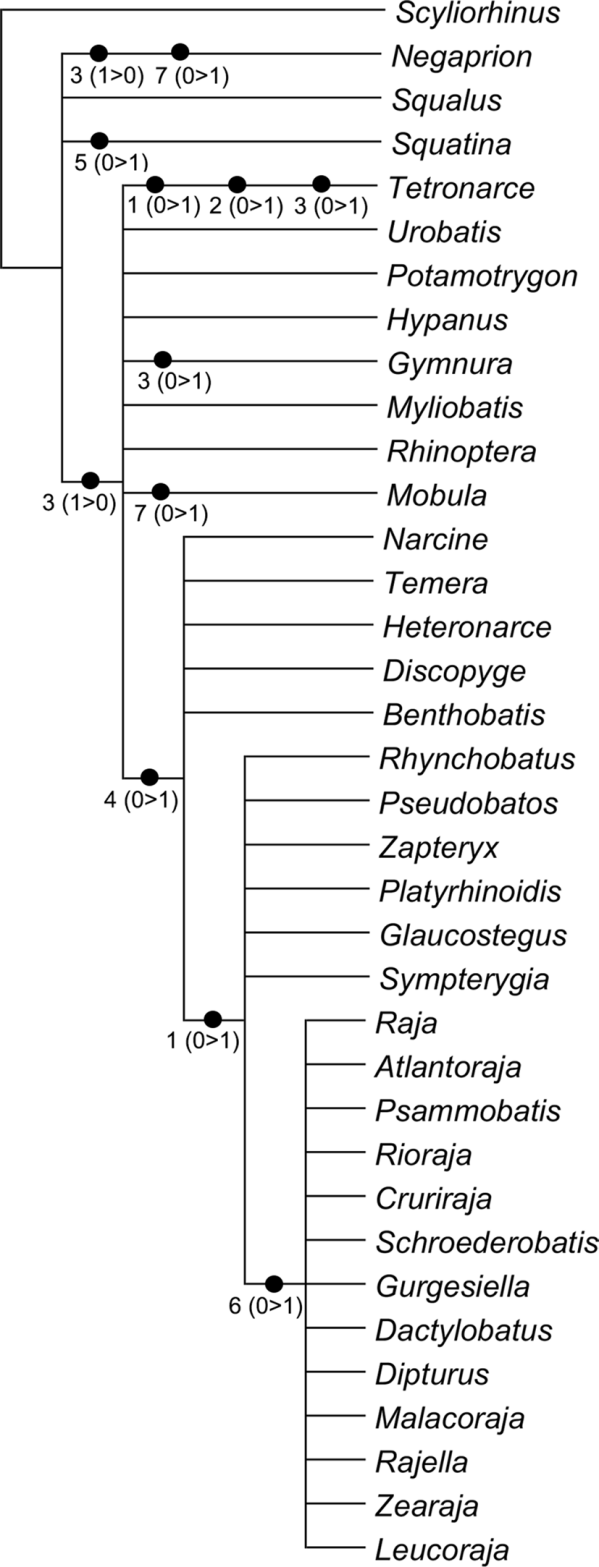
Strict consensus of the five equally most-parsimonious cladograms of 14 steps (CI = 0.71; RI = 0.91) resulted from the analysis including only characters from the afferent branchial arteries and character states changes.

The analysis considering only characters from the afferent branchial arteries resulted in five equally most-parsimonious cladograms of 14 steps (CI = 0.71; RI = 0.91). The strict consensus of those most parsimonious cladograms shows a monophyletic taxon that includes Rajiformes, Rhinobatiformes, Rhiniformes, Torpediniformes (except *Tetronarce*) and Platyrhinidae supported by the presence of a common trunk off which the three posteriormost afferent arteries branch (Fig. 9; ch. 4, state 1). Within this clade, the Rajiformes, Rhinobatiformes, Rhiniformes and Platyrhinidae are grouped on the basis of the derived presence of a long common trunk off the ventral aorta to the hyoidean and first branchial afferent arteries (ch. 1, state 1) and this condition is also present in *Tetronarce* (Fig. 9). Finally, all Rajiformes except *Sympterygia* form a monophyletic taxon supported by the derived condition of the second and third afferent branchial arteries splitting from a common branch (Fig. 9; ch. 6, state 1).

**Figure 10:**
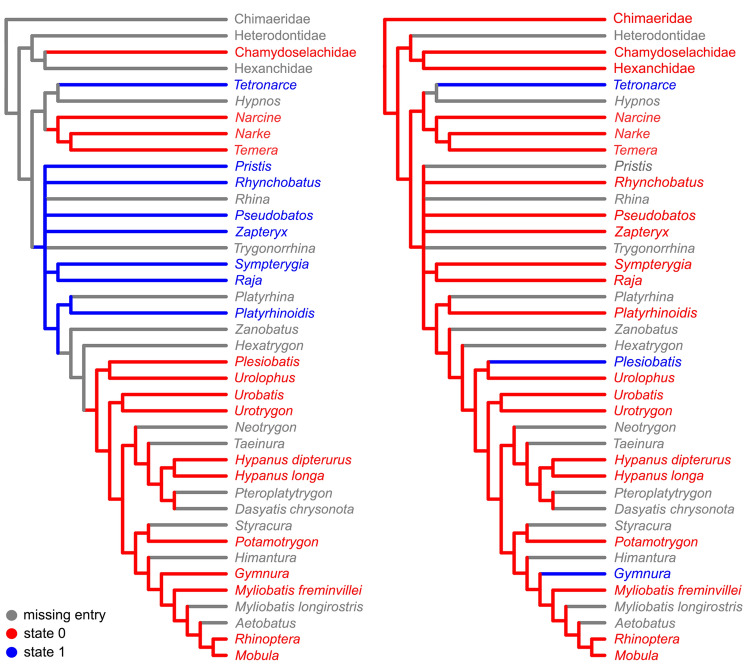
Distribution of characters 1 (left) and 3 (right) in the strict consensus of the two equally most-parsimonious trees of 211 steps (CI = 0.63; RI = 0.90) resulted from the analysis combining characters from the afferent branchial arteries with Aschliman’s et al.^3^ dataset.

**Figure 11:**
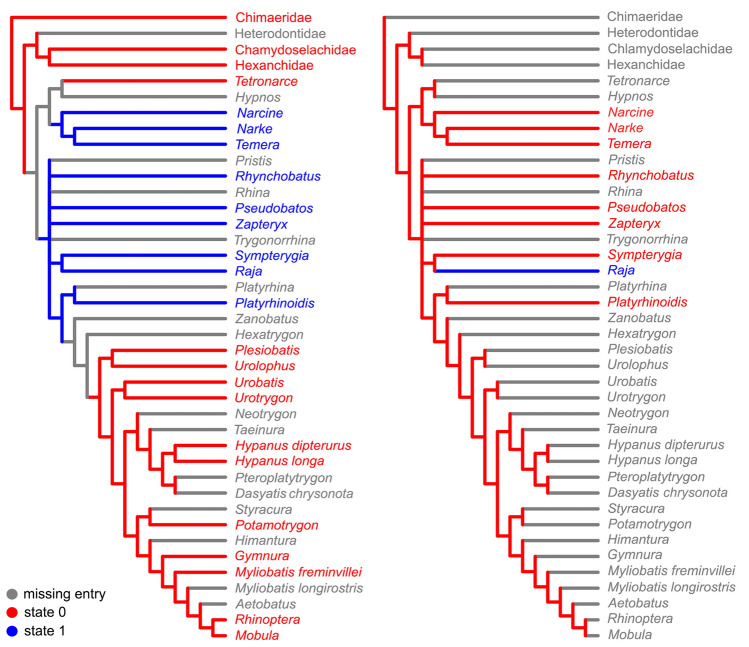
Distribution of characters 4 (left) and 6 (right) in the strict consensus of the two equally most-parsimonious trees of 211 steps (CI = 0.63; RI = 0.90) resulted from the analysis combining characters from the afferent branchial arteries with Aschliman’s et al.^3^ dataset.

The second analysis combining characters from the afferent branchial arteries with Aschliman et al.^3^ dataset resulted in two equally most-parsimonious cladrograms of 211 steps (CI = 0.63; RI = 0.90) and the strict consensus cladogram has the same topology as that presented by Aschliman et al.^3^ (p. 70, Fig. 3.7). The distribution of the characters from the afferent branchial arteries in the resulting topology are presented in Figs. 10 and 11.

## Discussion

The arrangement of afferent branchial arteries varies among batoids. Differences occur mainly at the level of orders and within these, variation at the level of families occurs only in a few cases (Table 1). Representatives of the four families of guitarfishes currently recognized show no variation for the characters examined. The fifteen species representing all four families of the Rajiformes also show no variation except for *Sympterygia* spp. which differs in one character-state (ch. 6, state 0 vs. 1 in other Rajiformes). Among the Torpediniformes, representatives of three out of four families were examined (all except the Hypnidae) and only the torpedinid *Tetronarce puelcha* differs in some of the characters examined (characters 1, 2 and 3). Representatives of seven out of the eleven currently recognized families of the Myliobatiformes were examined and variation was found in only two: the gymnurid *Gymura micrura* (ch. 3) and the mobulid *Mobula hypostoma* (ch. 7). Because we only examined one representative of the Torpedinidae, Gymnuridae and Mobulidae, it is not clear at this time if the different conditions we observed are restricted to the species examined or if they represent more general conditions at the genus or family level.

In addition to the morphological variation observed among taxa in the present study, different conditions of the afferent branchial arteries were reported for other chondrichthyans by previous authors. For instance, the hyoidean and first afferent branchial arteries branch independently from the ventral aorta in the sharks *Heptanchus cinereus* (= *Heptranchias perlo*)^33^ and *H*. *maculatus* (= *Notorynchus cepedianus*)^46^ and in the chimaera *Hydrolagus colliei*^35^, contrasting with the arrangement reported for most sharks and rays, in which those arteries originate from a common trunk at the anterior end of the ventral aorta (Figs. 1, 2, 3, 4, 5, 6, 7, 8; Muñoz-Chápuli^41^). Therefore, examination of additional taxa, in particular sharks, may show that variation in this character complex is greater than that reported in this study.

When analyzed on their own, characters from the afferent branchial arteries are not informative as to the question of monophyly of each of the orders Myliobatiformes, Torpediniformes, Rhinobatiformes and Rhiniformes (Fig. 9, Table 1), and one synapomorphy is herein proposed to support the monophyly of the Rajiformes. Among batoids that have a common trunk that branches into the three posteriormost afferent branchial arteries, the second and third afferent arteries split from a common branch (ch. 6, state 1) only in the Rajiformes (except *Sympterygia*). Illustrations provided by Fergunson^52^ (p.182, Fig. 7) and Allis^34^ (p. 584) depict the second, third and fourth arteries branching from the same point from the ventral aorta in *Raja erinacea* (= *Leucoraja erinacea*) and *Raja radiata* (= *Amblyraja radiata*), respectively, a condition which differs from that of congeners examined herein. In species of *Sympterygia* the three posteriormost afferent branchial arteries also split from a common trunk, but in those species, it is the third and fourth afferent arteries that originate from a common branch, an arrangement similar to that in *Platyrhinoidis triseriata*, Rhiniformes, Rhinobatiformes, and the torpediniforms Narcinidae and Narkidae. This condition is herein regarded as plesiomorphic for these taxa. In morphological^53^ and molecular^4,6^ analyses that investigated the interrelationships within Rajiformes, *Sympterygia* is placed deeply within the family Arhynchobatidae so that the presence of a common branch for the two posteriormost arteries in this genus would represent an autapomorphy.

The analysis including only characters from afferent branchial arteries group the Rajiformes with guitarfishes, on the basis of the presence of a long common trunk to the two anteriormost afferent arteries (ch. 1, state 1; Fig. 9), a feature that appears independently in the torpediniform *Tetronarce*. This condition seems to be also present in the sawfish *Pristis pectinatus* (= *Pristis pectinata*)^26^. A close relationship between those groups of rays was previously proposed by McEachran and Aschliman^20^. O’Donogue and Abbot^37^ suggested that the longer condition of the trunk off the ventral aorta for the hyoidean and first branchial afferent arteries could be a consequence of the flattening of the body, however representatives of Myliobatiformes and Torpediniformes (except *Tetronarce*) that have a flat body, possess a short trunk from the ventral aorta to the anteriormost afferent arteries. Kobelkowsky^43^ proposed a close association between the length of the common trunk of the two anteriormost afferent arteries and the width of the *coracohyomandibularis* muscle. However, shark taxa that present a long trunk lack a *coracohyomandibularis* muscle. The position of the branching point of the hyoidean and first afferent branchial arteries could be related to the occurrence and relative width of the basihyal cartilage, which is wide and arched in Platyrhinidae, Rajiformes, Rhiniformes and Rhinobatiformes, although absent in *Tetronarce*^54^. Among sharks that present a long trunk, the basihyal is also wide in *Pristiophorus* but relatively narrow in *Squatina*^54^.

Among examined elasmobranchs, the Rajiformes, guitarfishes and the Torpediniformes (except *Tetronarce*) uniquely share the presence of a common trunk that branches into the three posteriormost afferent branchial arteries (ch. 4, state 1; Fig. 9), a feature previously proposed by Corrington^38^ and Daniel^46^ as the “general pattern of batoids”. The second, third and fourth afferent branchial arteries splitting from a common trunk was illustrated for *Tetronarce nobiliana* (Satchell^42^; p. 220, Fig. 9.1) and *Torpedo* sp. (Muñoz-Chápuli^41^; p. 199, Fig. 8.1), but according to our observations of *T. puelcha*, only the third and fourth afferent arteries branch from a common trunk, with the second afferent artery branching independently from the ventral aorta. The illustration provided by Satchell^42^ was schematic, and the condition in the species could be inaccurately represented or alternatively, there could be variation in the condition of those vessels within the Torpedinidae. In the Myliobatiformes (except *Gymnura*) the second, third and fourth branchial afferent arteries branch independently from the ventral aorta, a condition also reported for *Dasyatis sabina*, *Myliobatis* sp., *Urobatis jamaicensis*, *Urotrygon munda* and *Urolophus paucimaculatus*^43,54^.

*Tetronarce* and *Gymnura* possess a common trunk off the ventral aorta for the third and fourth arteries (ch. 3, state 1), a feature also described for the myliobatiform *Urotrygon daviesi* (= *Plesiobatis daviesi*)^54^. This condition is also present among the examined sharks, *Scyliorhinus*, *Squalus* and *Squatina* and it was previously reported for *Mustelus antarcticus*, *Chilloscyllium modestum* (= *Brachaelurus waddi*) and *Centrophorus calceus* (= *Deania calceus*)^28,30,31^. According to O’Donoghue and Abbot^37^ having the two posteriormost arteries branching separately from the ventral aorta is the most common condition among sharks and is present in *Negaprion*, the hexanchiforms *Heptanchus cinereus* (= *Heptranchias perlo*), *Notorynchus* and *Chlamydoselachus*, the carcharhiniform *Galeus glaucus* (= *Prionace glauca*), *Cestracion zygaena* (= *Sphyrna zygaena*), and the lamniform *Carcharias littoralis* (= *Odontaspis taurus*)^33,38,46^. De Beer^55^ (p. 52, Fig. 27) illustrated the third and fourth arteries branching directly from the ventral aorta and well-spaced from each other in adult specimens of *Scyliorhinus canicula*, an observation that differs from that of the specimen we examined.

A long common trunk from which the third and fourth afferent arteries branch is observed only in *Squatina* among sharks and batoids examined (ch. 5, state 1) and is interpreted as an autapomorphy for this genus (Figs. 7, 9). A similar condition seems to be present in *Chiloscyllium modestum* (= *Brachaelurus waddi*)^30^ but needs confirmation.

*Tetronarce puelcha* shows a unique condition in comparison to all examined batoids in which the ventral aorta is distinctly short with the branching point of all vessels from the ventral aorta next to each other (ch. 2, state 1). A similar condition was also described for *T. californica*^26^ and *T. nobiliana*^42^ and is herein proposed as an autapomorphy for this genus. Among Torpediniformes, O’Donogue and Abbot^37^ emphasized the need to consider the ontogeny of afferent branchial arteries in chondrichthyans in order to understand their homologies. Thus, investigation of the ontogeny of all afferent branchial arteries in more chondrichthyans may help to better understand the nature of the variation observed in the taxa mentioned above.

A well-developed cranial coronary artery is present in *Mobula hypostoma* and *Negaprion brevirostris* but not in other taxa examined; such condition is proposed as an autapomorphy for each species. Davie and Farrel^51^ pointed out that a coronary supply is observed in active swimming fishes accompanied by a higher blood pressure and a relatively larger heart. The lesser devil ray *Mobula hypostoma* and the lemon shark *Negaprion brevirostris* inhabit coastal waters from Western Atlantic and are known to swim great distances^56–59^. However, a cranial coronary artery was not found in the pelagic stingrays *Myliobatis fremminvilei* and *Rhinoptera bonasus*.

The inclusion of characters from the afferent branchial arteries in the more comprehensive analysis of Aschliman et al.^3^, did not change the topology of their original cladogram, but the interpretation of the variation of character states of this anatomical complex differs from the analysis in which we only considered these characters (Figs. 10, 11). In Aschliman et al.^3^’s analysis, the order Torpediniformes is the sister taxon of all other batoids, with guitarfishes, Rajiformes and a clade with remaining batoids forming an unresolved polytomy. Within this latter clade the order Myliobatiformes is supported as a monophyletic taxon derived within Batoidea in most morphological and molecular phylogenetic inferences^2–7,11,15,18,20–24^. Under this phylogenetic hypothesis, the presence in Myliobatiformes of a short trunk to the two anteriormost afferent arteries (ch. 1, state 0) and the absence of a common trunk to the three posteriomost arteries (ch. 4, state 0) is a synapomorphic condition and would support the monophyly of this taxon (pending examining the condition in *Hexatrygon* and *Zanobatus*) (Figs. 10, 11).

Within Myliobatiformes, the presence of a trunk to the third and fourth arteries in *Gymnura* and *Plesiobatis* (ch. 3, state 1, Fig. 10) could be interpreted as autapomorphic conditions of both genera in the hypotheses of Aschliman et al.^3^ and Shirai^2^ as well as in the molecular hypotheses of Naylor et al.^4,5^. In the most recent analysis of Naylor^6^, the interpretation of the presence of such a trunk is ambiguous and could represent either a synapomorphy for the clade formed by Gymnuridae, Plesiobatidae and Urolophidae with a secondary modification in the latter family or as autapomorphies for *Gymnura* and *Plesiobatis*. A common trunk to the two posteriormost arteries is interpreted as an autapomorphy for the torpediniform *Tetronarce* in the hypothesis of Aschliman et al.^3^ (Fig. 10) and could represent a synapomorphy of the Torpedinidae if the condition in *Torpedo* is confirmed.

The arrangement of the afferent branchial arteries in the single representative of Platyrhinidae examined herein is very similar to that of the other guitarfishes (Table 1), which is characterized by a relatively long common trunk to the hyoidean and first afferent arteries, the three posteriormost afferent branchial arteries branching from a common trunk with the third and fourth arteries splitting from a common branch. Those conditions support the placement of *Platyrhinoidis* together with guitarfishes and the Rajiformes when characters from afferent branchial arteries are analyzed separately. A close relationship between Platyrhinidae and rhinobatids was already hypothesized by Shirai^2,7^ and Nishida^18^ while other morphological hypotheses recovered this family as closely related to *Zanobatus* + Myliobatiformes^3,20,22^. Alternatively, recent molecular hypotheses have placed Platyrhinidae in a close relationship with the Torpediniformes^4–6,11^. *Zanobatus* was not examined here and further work is needed to resolve the placement of this taxon and Platyrhinidae within Batoidea.

Although the present study has expanded considerably the information available about variation in the afferent branchial arteries in batoids, the analysis of the characters proposed in the more comprehensive analysis of Aschliman et al.^3^ shows that the lack of information about the variation of characters from the afferent branchial arteries in many batoid genera and in most shark and holocephalan families results in ambiguities in many nodes. The coding of character states for those taxa may provide a more complete understanding of the evolution of this character complex in Chondrichthyans, however conflict may also still remain. Moreover, given the amount of divergence and conflict in phylogenetic hypotheses of elasmobranchs based on different sets of characters and taxa examined by different authors, the distribution of character states could be interpreted differently depending on the phylogenetic hypotheses considered. For instance, among sharks, *Squalus* and *Squatina* possess a long common trunk to the hyoidean and first afferent branchial arteries (ch. 1, state 1), a condition also present in *Pristiophorus japonicus* (based on Miyake et al.^26^, Fig. 19). Given the distribution of this condition among elasmobranchs, a long trunk could be interpreted as a synapomorphy for the clade formed by squalids, squatinids, pristiophorids and batoids in the morphological hypotheses of Shirai^2,7^ which resolved batoids as derived sharks. Alternatively, the same condition could be independently acquired in the clade formed by Squatiniformes and Pristiophoriformes, in *Squalus* and in Batoidea or represent a plesiomorphic condition for elasmobranchs according to the molecular hypotheses of Naylor et al.^4–6^ that recovered the reciprocal monophyly of sharks and rays. In the same way, the presence of a trunk from which the three posteriormost afferent arteries branch (ch. 4, state 1) has an ambiguous optimization for Batoidea in the hypothesis of Aschliman et al.^3^ and could be interpreted either as synapomorphic for Batoidea or as independent acquisitions of Narcinidae, Narkidae, and the clade formed by guitarfishes, Rajiformes and Myliobatiformes (Fig. 11). In hypotheses that place guitarfishes or Rajiformes at a basal position within Batoidea^2–7^, this common vessel would represent a putative synapomorphy for the superorder with reversal to the more primitive condition of lacking a common trunk to the three posteriormost afferent arteries in *Tetronarce* and in the Myliobatiformes.

## Conclusions

Characters from the branching patterns of the afferent branchial arteries proposed herein were analyzed on their own and integrated in the most recent morphological hypothesis on the interrelationships of Batoidea. Although a complete understanding of the evolution of the branching patterns of the afferent branchial arteries in chondrichthyans depends on the examination of additional taxa, mainly Squaliformes and Chimaeriformes, from the analyses carried out in the present study, we conclude that:

- The branching pattern of afferent branchial arteries varies among batoid orders and families and is potentially informative for batoid systematics;
- Among taxa that have a common trunk from the ventral aorta from which the three posteriormost afferent arteries branch, having a common branch to the second and third arteries is a synapomorphy for Rajiformes with a reversal to the condition in which the third and fourth arteries split from a common branch interpreted as autapomorphic for *Sympterygia*;
- The presence of a well-developed cranial coronary artery is an autapomorphy for *Mobula hypostoma* and *Negaprion brevirostris*;
- The torpediniform *Tetronarce* has a unique condition in comparison to all examined batoids and sharks in which the ventral aorta is distinctly short with the branching point of all vessels from the ventral aorta in close proximity to each other. This condition is autapomorphic for the genus and an ontogenetic study of afferent branchial arteries in Torpedinidae could help to better understand the nature of the variation observed;
- Although the presence of a common trunk off the ventral aorta from which the three posteriormost afferent arteries branch is uniquely observed in batoids, the conclusion that this condition is synapomorphic for Batoidea depends on the resolution of ambiguities at the base of this clade, which could be resolved by the examination of additional taxa (e.g., *Hypnos*) or after current conflicts about the interrelationships within batoids are clarified. In any case, given congruence among different hypotheses of batoid relationships regarding the derived position of the Myliobatiformes, the condition in which the second afferent branchial artery branches directly from the ventral aorta, independently from the last two afferent arteries is a synapomorphy for the order.

## Material and methods

All specimens examined herein were obtained from museum collections. Thirty-three specimens belonging to 32 genera of rays were examined, comprising 19 of the 26 families currently valid for the superorder Batoidea^44^. Classification for batoid families and genera follows Last et al.^44^ except for guitarfishes and Platyrhinidae. The order Rhinopristiformes was proposed by Naylor et al.^4^, on the basis of analysis of molecular data, to include sawfishes (Pristidae) and guitarfishes. However, this taxon was never recovered as monophyletic in phylogenetic analyses based on morphological characters^2,3,7,20,22^. In the present study we did not examine representatives of sawfishes and chose to follow Weigmann^45^, when referring to guitarfishes as representatives of the orders Rhinobatiformes and Rhiniformes. The phylogenetic relationship of the Platyrhinidae is controversial and the family has already been proposed as closely related to rhinobatiforms^2^, myliobatiforms^3,20,22^ or torpediniforms^5,6^. Herein we considered it as ‘*incertae sedis*’. The shark genera *Negaprion, Scyliorhinus* (superorder Galeomorphi)*, Squalus* and *Squatina* (superorder Squalomorphi) were also examined as comparative taxa.

Specimens examined are deposited in the following institutions: CAS, California Academy of Sciences, San Francisco; MCP, Museu de Ciências e Tecnologia, Pontifícia Universidade Católica do Rio Grande do Sul, Porto Alegre; MCZ, Museum of Comparative Zoology, Cambridge; MNRJ, Museu Nacional, Rio de Janeiro; MZUSP, Museu de Zoologia da Universidade de São Paulo, São Paulo; UERJ, Universidade do Estado do Rio de Janeiro, Rio de Janeiro; USNM, National Museum of Natural History, Smithsonian Institution, Washington D.C. Specimens examined are listed below. The abbreviation TL used throughout the text refers to total length.

### Myliobatiformes

Dasyatidae: *Hypanus marianae*, MZUSP 52885, female, 427 mm TL (Abrolhos, Bahia, Brazil). Gymnuridae: *Gymnura micrura*, MZUSP 122987, female, 255 mm TL (Caiçara do Norte Beach, Rio Grande do Norte, Brazil, 5° 3′45″ S, 36°3′45″ W). Myliobatidae: *Myliobatis freminvillei*, MZUSP 9927, female, 672 mm TL (Rio Grande do Sul, Brazil, 29°23′ S, 49°16′ W). Mobulidae: *Mobula hypostoma*, MZUSP 59291, male, 751 mm TL (Guarujá, São Paulo, Brazil). Potamotrygonidae: *Potamotrygon motoro*, MZUSP 82468, female, 420 mm TL (Tapajós River, Pará, Brazil, 2°24′46″ S, 54°53′30″ W). Rhinopteridae: *Rhinoptera bonasus*, MZUSP 72932, male, 568 mm TL (Barequeçaba Beach, São Paulo, Brazil). Urotrygonidae: *Urobatis halleri*, USNM 181332, male, 315 mm TL (Isla Santa Margarita, Baja California, Mexico).

### Rajiformes

Anachantobatidae: *Schroederobatis americana*, MCZ 42953, female, 306 mm TL (French Guiana). Arhynchobatidae: *Atlantoraja cyclophora*, MZUSP 117184, female, 583 mm TL (Itajaí, Santa Catarina, 26°41’S, 46°42’’W); *Psammobatis extenta*, MNRJ 32468, female, 370 mm TL (Rio de Janeiro, Brazil); *Rioraja agassizii*, MNRJ 50512, female, 505 mm TL (Rio de Janeiro, Brazil, 23°11′30″ S, 42°58′30″ W); *Sympterygia acuta*, MCP 4745, male, 525 mm TL (Rio Grande do Sul, Brazil, 32°14’S, 52°6′60’’W), *S. bonapartii*, MZUSP 11781, female, 416 mm TL (Santa Catarina, Brazil, 27º38′52″ S, 48º20′16″ W). Gurgesiellidae: *Cruriraja rugosa*, MCZ 41970, female, 318 mm TL (Caribbean Sea); *Gurgesiella atlantica*, MNRJ 9535, female, 370 mm TL (Suriname, 7°15’N, 53°21’W). Rajidae: *Dactylobatus clarkii*, MCZ 51810, male, 369 mm TL (Magdalena, Colombia); *Dipturus* sp., UERJ E505, male, 426 mm TL (no locality data); *Leucoraja garmani*, MCZ 37162, female, 317 mm TL (Gay Head, Massachusetts, United States); *Malacoraja senta*, MCZ 37091, male, 284 mm TL (New England, United States); *Raja miraletus*, USNM 193737, male, 408 mm TL (Liberia); *Rajella purpuriventralis*, UERJ D506, female, 390 mm TL (São Paulo, Brazil); *Zearaja chilensis*, MCP 3756, male, 278 mm TL (Uruguay).

### Rhiniformes

Rhinidae: *Rhynchobatus palpebratus*, MZUSP 125618, male, 576 mm TL (Queensland, Australia, 12°24′3″ S, 12°25′9″ E).

### Rhinobatiformes

Glaucostegidae: *Glaucostegus granulatus*, USNM 149733, male, 339 mm TL (India). Rhinobatidae: *Pseudobatos horkelii*, MNRJ 51463, female, 652 mm TL (Rio de Janeiro, Brazil, 21°36′58″ S, 41°2′55″ W). Trygonorhinidae: *Zapteryx brevirostris*, MZUSP 117272, 455 mm TL (Ubatuba, São Paulo, Brazil).

### Torpediniformes

Narcinidae: *Benthobatis kreffti*, MZUSP 86555, female, 264 mm TL (Brazil, 25°46′11″ S, 45°11′49″W); *Discopyge tschudii*, MZUSP 72780, female, 291 mm TL (Uruguay, 35°18′ S, 54°13′ W); *Narcine brasiliensis*, MZUSP 72799, male, 263 mm TL (Peruíbe, São Paulo, Brazil). Narkidae: *Heteronarce* sp., CAS 58351, female, 232 mm TL (Somalia); *Temera hardwickii*, CAS 35736, 122 mm TL (Singapore). Torpedinidae: *Tetronarce puelcha*, MZUSP 86769, male, 296 mm TL (Brazil, 23°44′17″ S, 42°12″ W).

### Incertae sedis

Platyrhinidae: *Platyrhinoidis triseriata*, CAS 59621, male, 383 mm TL, CAS 31248, female, 313 mm T (United States).

### Comparative material

*Negaprion brevirostris*, MNRJ 16588, male, 625 mm TL (Atol das Rocas, Rio Grande do Norte, Brazil); *Scyliorhinus haeckelii*, UERJ 2209, male, 461 mm TL (between Rio de Janeiro and São Paulo states, Brazil); *Squalus* sp., USP uncatalogued, 4 specimens (no locality data); *Squatina occulta,* MZUSP 42851, female, 532 mm TL (Brazil, 28°43′ S, 48°20′ W).

Descriptions of the heart and afferent branchial arteries were based on specimens preserved in 70% ethanol and dissected with the aid of forceps, scalpels and fine-tipped scissors. The skin and hypobranchial musculature were removed to expose the heart, the ventral aorta and afferent branchial arteries, which were removed together with and stored in 70% ethanol. Photographs were taken with a Canon Power Shot SX610 HS and edited in Adobe Photoshop CS6.

Some authors refer to the vessels that originate from each side of the anterior end of the ventral aorta and split into the hyoidean and the first afferent branchial arteries as the anterior innominate arteries (*e.g*.^29,30,36–38,42,43^), but others (*e.g*.^26,32–35,46^) use more general, descriptive terms such as “common trunks” or “common vessels” of the hyoidean and first afferent branches of the ventral aorta. In a similar way, the term “posterior innominate arteries” is used for the vessels that originate from the ventral aorta and split into the third and fourth or into the second, third and fourth afferent branchial arteries. Using the same term to refer to a common vessel from which two vessels branch off and from which three vessels branch off raises the question of homology of the common vessel in those two cases. Therefore, in the present study we do not use the term “posterior innominate” and refer to these vessels as common trunk of the third and fourth afferent branches of the ventral aorta or common trunk of the second, third and fourth afferent branches of the ventral aorta. For consistency, we also chose not to use the term “anterior innominate”. We followed the terminology used by Allis^32–35^ for the afferent branchial arteries and refer to the artery associated with the hyoid arch as the hyoidean artery and to the arteries associated with the first, second, third and fourth branchial arches as first, second, third and fourth afferent branchial arteries, respectively. The term “ventral aorta” follows Parker^28^ and Corrington^38^.

The characters proposed were tested in two different analyses. The first included all 36 genera of examined batoids and sharks and seven characters related to the branching patterns of the afferent branchial arteries. The monophyly of Batoidea was forced a priori by adding three random characters, so we could observe the changes in the branching patterns of the afferent arteries within this clade. *Scyliorhinus* was chosen to root the cladogram because this taxon is included in the superorder Galeomorphi, a clade phylogenetically distant from Batoidea and for its basal placement within carcharhiniforms in all morphological and DNA-based phylogenetic studies.

In the second analysis, characters related to the branching patterns of the afferent branchial arteries were concatenated in the data matrix of Aschliman et al.^3^ which included 36 batoids and 4 outgroups (one holocephalan and three sharks) and 89 characters and which is the most recent analysis on the interrelationships within Batoidea based on morphological characters. Because we did not examine the same species analyzed by Aschliman et al.^3^, only supraspecific taxa were considered as terminal taxa in our analysis with the exception of the species of *Dasyatis, Hypanus* and *Myliobatis*. Since all examined Rajiformes (except *Sympterygia*) present the same branching pattern for the afferent branchial arteries, they were merged into the terminal ‘*Raja’* of Aschliman et al.^3^. To express the differences between *Sympterygia* and the other Rajiformes regarding the afferent branchial arteries, the terminal ‘*Bathyraja’* of Aschliman et al.^3^ matrix was replaced by *Sympterygia* since both genera belong to the family Arhynchobatidae. Shark (*Negaprion*, *Scyliorhinus*, *Squalus* and *Squatina*) and batoid (*Glaucostegus*, *Benthobatis, Discopyge* and *Heteronarce*) taxa examined herein that were not part of the study of Aschliman et al.^3^ were not included to avoid missing entries in characters related to other anatomical complexes in the data matrix. Character 5 of the present study refers to a unique condition of *Squatina* and since this taxon was not examined by Aschliman et al.^3^, this character was excluded from this analysis, and only six characters related to the branching patterns of the afferent branchial arteries (1–4, 6, 7) were considered. The combined data matrix included a total of 40 taxa and 95 characters. Available data in the literature about the branching patterns of the afferent branchial arteries in *Hydrolagus*^35^, *Chlamydoselachus*^29,32^ and *Heptranchias*^33^ were incorporated in the terminal taxa ‘Chimaeridae’, ‘Chlamydoselachidae’ and ‘Hexanchidae’ of Aschliman et al.^3^ matrix, respectively. In addition, available data for the genera *Plesiobatis*, *Pristis*, *Urotrygon* and *Urolophus* were also included^26,54^. Character states of terminal taxa that were not examined or were inapplicable were coded as (–).

Maximum parsimony analyses were performed in TNT 1.5 (Goloboff et al.^47^) for the two data matrices mentioned above, using a heuristic search option and tree-bisection-reconnection (TBR) algorithm with random sequence addition of 100 replicates per search and retained trees limit set to 5000 (following the parameters of Aschliman et al.^3^). All characters were analyzed as equally weighted and unordered. Strict consensus trees were built through TNT. Character optimizations were obtained in the software WINCLADA version 1.0^48^ using the option ‘unambiguous changes only’. Tree edition was performed with the aid of WINCLADA and Adobe Photoshop CS6.

## Data Availability

All data generated or analyzed during this study are included in this published article.
